# Thermal Pre-Aging-Dependent Seawater-Induced Degradation of XLPE Submarine Cable Insulation: Electrical Performance Evolution and Microstructural Mechanisms

**DOI:** 10.3390/polym18141747

**Published:** 2026-07-16

**Authors:** Liang Zou, Shoushui Han, Zhiyun Han, Rongzhao Jia, Qingsong Liu, Zheng Liu, Hanwen Ren

**Affiliations:** 1 School of Electrical Engineering, Shandong University, Jinan 250061, China; zouliang@sdu.edu.cn (L.Z.); 202434730@mail.sdu.edu.cn (S.H.); hanzhiyun@sdu.edu.cn (Z.H.); 202334730@mail.sdu.edu.cn (Z.L.); 2 Research Institute of Electric Power, China Southern Power Grid Ultra-High Voltage Transmission Company, Guangzhou 510663, China; didovo@163.com; 3 State Key Laboratory of Alternate Electrical Power System with Renewable Energy Sources, North China Electric Power University, Beijing 100096, China; rhwncepu@ncepu.edu.cn

**Keywords:** cross-linked polyethylene, thermal aging, seawater corrosion, submarine cable, time-series coupling

## Abstract

The long-term reliability of XLPE submarine cable insulation is influenced by progressive thermal degradation during operation and subsequent seawater ingress caused by external damage. Although thermal aging and seawater exposure have been widely investigated individually, the influence of the prior thermal-aging state on the subsequent seawater-induced degradation behavior of XLPE remains insufficiently understood. In this study, XLPE insulation specimens prepared from the same commercial compound used for 500 kV submarine cables were subjected to sequential accelerated aging consisting of controlled thermal pre-aging followed by simulated seawater exposure. Broadband dielectric spectroscopy, AC breakdown testing with two-parameter Weibull analysis, scanning electron microscopy (SEM), and Fourier-transform infrared spectroscopy (FTIR) were employed to investigate the evolution of electrical properties, surface morphology, and molecular structure. The results demonstrate that seawater-induced electrical deterioration strongly depends on the initial thermal-aging state of XLPE. Increasing thermal pre-aging duration resulted in progressively higher relative permittivity and dielectric loss, together with reduced characteristic breakdown strength after subsequent seawater exposure. Under the most severe condition of 1440 h thermal pre-aging followed by 672 h seawater exposure, the power–frequency relative permittivity increased by 32.1%, while the characteristic breakdown strength decreased by more than one-third compared with the initial state. SEM observations revealed that thermally pre-aged specimens developed accelerated surface damage during seawater exposure, including pores, cracks, corrosion pits, and honeycomb-like structures. FTIR analysis further indicated molecular-chain degradation and increased hydroxyl-related species during sequential aging. These results suggest that thermal-aging-induced molecular oxidation, polar-group formation, and microstructural defects enhance water and ion penetration pathways, thereby increasing the susceptibility of XLPE insulation to subsequent seawater-induced degradation. This study provides material-level experimental evidence for understanding sequential aging processes in submarine cable insulation and highlights the importance of considering historical thermal damage in future condition assessment and lifetime evaluation models. Since accelerated laboratory conditions were adopted, the results should be interpreted as comparative degradation characteristics rather than direct predictions of field-service lifetime.

## 1. Introduction

With the rapid development of offshore wind power and integrated land–sea power systems, submarine cables have become indispensable infrastructure for long-distance power transmission and offshore renewable-energy integration [1,2,3]. Among various insulation materials, cross-linked polyethylene (XLPE) has become the mainstream insulation material for high-voltage and extra-high-voltage submarine cables because of its excellent dielectric properties, thermal stability, environmental compatibility, and ease of manufacture [4]. Nevertheless, the long-term operational reliability of XLPE submarine cables remains one of the key factors affecting the security and stability of offshore power systems [5]. Statistics indicate that insulation degradation caused by long-term service and external damage is one of the primary causes of submarine cable failure, leading to substantial economic losses and maintenance costs [6].

Previous studies have extensively investigated the degradation behavior of XLPE insulation under individual environmental stresses. Thermal aging has been shown to induce molecular-chain scission, oxidation, crystallinity variation, and microstructural defects, resulting in deterioration of both dielectric and mechanical properties [7,8,9,10,11]. In addition to thermal aging, external mechanical damage is another major threat to submarine cable reliability. Once the outer sheath or metallic armor is damaged by anchors, fishing activities, or ship dragging, seawater can directly penetrate the insulation layer, accelerating electrical degradation through water uptake and ion transport [12,13,14,15]. Existing studies have demonstrated that seawater exposure promotes defect evolution and dielectric deterioration in XLPE insulation, while several investigations have also discussed the influence of service conditions and external loading on submarine cable reliability [16,17,18].

Despite these important advances, most existing studies investigate thermal aging and seawater exposure as independent degradation processes. However, the degradation history experienced by practical submarine cables is fundamentally different. During long-term operation, XLPE insulation continuously accumulates thermo-oxidative damage because of conductor heating. Only after external mechanical damage or sheath failure does seawater penetrate into the already aged insulation. Consequently, the degradation process follows a sequential path in which seawater exposure acts on insulation that has already undergone different degrees of thermal-aging-induced pre-damage. This service-oriented degradation sequence has received considerably less attention than isolated thermal-aging or seawater-corrosion studies. Furthermore, although several studies have reported changes in dielectric properties or surface morphology under individual aging conditions, relatively little information is available regarding how the prior thermal-aging state modifies the subsequent response of XLPE to seawater exposure. The interaction between thermal-aging-induced defects and subsequent seawater ingress therefore remains insufficiently understood, particularly from the perspective of electrical insulation performance and microstructural evolution.

To address this research gap, this study establishes a sequential accelerated-aging procedure in which XLPE insulation specimens are first subjected to controlled thermal pre-aging and subsequently exposed to simulated seawater. Broadband dielectric spectroscopy, AC breakdown testing combined with Weibull statistical analysis, SEM observation, and FTIR characterization are integrated to investigate the evolution of electrical performance, surface morphology, and molecular structure under different thermal pre-aging states. Unlike previous studies that mainly focus on isolated thermal aging or seawater exposure, this work specifically examines how thermal-aging-induced pre-damage modifies the subsequent degradation sensitivity of XLPE insulation during seawater exposure. The objectives of this study are threefold: (1) to clarify the dependence of seawater-induced dielectric and breakdown degradation on different levels of prior thermal aging; (2) to reveal the relationship between electrical performance deterioration and surface/molecular structural evolution; and (3) to propose a material-level degradation mechanism describing how thermal oxidation, defect formation, and ion penetration interact during sequential aging.

It should be noted that this study does not aim to reproduce the complete service environment of submarine cables or establish a direct lifetime prediction model. Instead, it provides comparative experimental evidence for understanding the degradation pathway of XLPE insulation under sequential thermal and seawater stresses, which may contribute to future condition assessment and lifetime modeling of submarine cable insulation systems.

## 2. Design of a Stepwise Accelerated Aging Test for XLPE Submarine Cable Insulation Subjected to Thermal Aging and Seawater Corrosion

### 2.1. Preparation of XLPE Insulation Test Specimens

The insulating specimens used in this study were prepared from the same commercial XLPE insulation compound used for practical 500 kV submarine cables. The compound contained the polyethylene base resin together with the industrial peroxide crosslinking system and hindered phenolic antioxidant package used in cable insulation manufacture. Therefore, the present specimens are representative of the practical insulation material at the material-composition level rather than being prepared from pure polyethylene resin.

It should be noted that the compression-molded specimens used in this study do not completely reproduce the extrusion-induced morphology of industrial submarine cable insulation. The extrusion process may introduce molecular-chain orientation, radial crystallinity variation, and residual mechanical stress due to continuous cooling and crosslinking procedures. Therefore, the present specimen preparation method was adopted to obtain homogeneous XLPE samples and minimize structural variables associated with cable manufacturing processes.

Nevertheless, the laboratory specimens do not reproduce the complete multilayer structure of practical submarine cables, including semiconductive screens, metallic conductors, extrusion-induced morphology, radial crystallinity gradients, residual stresses, or the coupled thermal–electrical environment during service. Therefore, the conclusions should be interpreted as material-level degradation characteristics of XLPE insulation rather than the complete aging behavior of full-scale submarine cables.

The specimens were prepared from the commercial XLPE insulation compound rather than pure polyethylene resin. The objective of this work is to investigate the intrinsic degradation interaction between thermal pre-aging and subsequent seawater exposure at the XLPE material level. Therefore, the conclusions are mainly applicable to the degradation tendency and mechanism of XLPE insulation materials rather than the exact structural evolution of a complete submarine cable.

**Figure 1:**
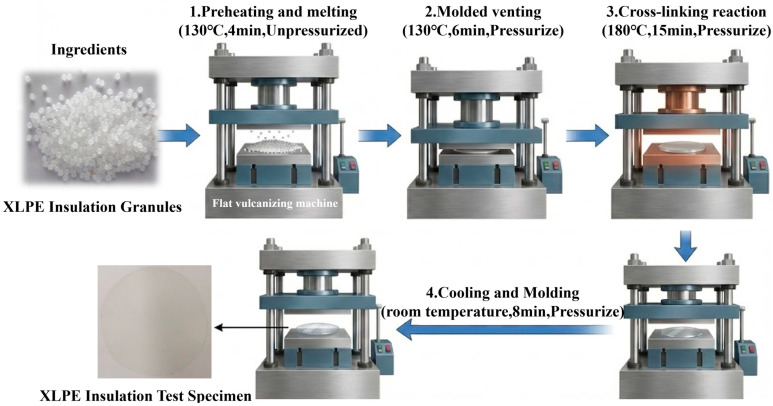
XLPE insulation sample preparation process.

The raw materials were mainly composed of low-density polyethylene resin, diisopropylbenzene peroxide cross-linking agent, and hindered phenolic antioxidants. Given that mechanical damage and local stress concentration were easily introduced on the specimen surface by the slicing method, the high-temperature compression molding method was adopted for specimen preparation in this experiment [19]. The preparation process was carried out on a flat-plate vulcanizer. Staged temperature and pressure control was applied to ensure uniform internal cross-linking and crystalline structure of the specimens. The specific process was illustrated in Figure 1.

The granular XLPE was first pretreated for 24 h in a vacuum oven at 70 °C to remove moisture and volatile impurities adsorbed during storage and transportation, thereby preventing the formation of microporosity or voids within the specimen during molding. The dried raw material was evenly spread into a stainless steel mold preheated to 130 °C. It was preheated for 4 min under no pressure to allow the granules to fully melt and transition to a viscous flow state, ensuring good flowability for subsequent molding. Subsequently, a pressure of 5 MPa was applied at 130 °C and maintained for 6 min. In conjunction with mold venting, residual bubbles in the melt were expelled, completing the initial molding. The mold temperature was then raised to 180 °C, and the pressure was increased to 15 MPa and maintained for 15 min to ensure complete cross-linking of the material. After molding, the specimens, along with the mold, were rapidly transferred to a water-cooled press and cooled to room temperature at a rate of 10 °C/min under 15 MPa pressure, ultimately yielding circular specimens with a uniform thickness of 0.3 mm and a diameter of 100 mm. After sample preparation, all specimens were placed in a vacuum oven at 8 °C for 24 h prior to formal testing to release residual thermal stress and remove volatile byproducts generated during the cross-linking process, ensuring consistent initial conditions for the experiment.

### 2.2. Multi-Gradient Thermal Aging Pre-Damage Acceleration Test

Determining a reasonable thermal aging temperature was central to the design of the accelerated aging test scheme. The key lay in achieving an optimal balance between accelerated degradation efficiency and consistency with the actual failure mechanism, while ensuring that the test operating conditions corresponded to those of subsequent seawater corrosion tests. Based on the maximum operating temperature of XLPE submarine cables, the maximum steady-state temperature of the XLPE cable conductor core can reach 90 °C. According to the laws of heat conduction in cylindrical bodies, the thermal stress level sustained by the inner insulation layer immediately adjacent to the conductor over the long term is extremely close to the conductor temperature [20]. If the aging temperature is set too high, although the test duration can be significantly shortened, severe thermal cracking or even combustion of the polymer matrix is highly likely to be induced, deviating from the thermal-oxidative aging mechanism under actual service conditions.

Taking into account both the actual temperature rise characteristics of submarine cables during service and the physical phase transition features of XLPE materials, the thermal aging temperature was set at 130 °C in this study. Although this temperature is slightly higher than the crystal melting peak, it does not completely unbind the polymer chains; the material retains a certain degree of structural memory, ensuring that the aging mechanism remains primarily driven by thermal oxidation reactions.

The thermal-aging temperature was set to 130 °C based on the thermal characteristics of the XLPE insulation compound. According to manufacturer data, the crystalline melting peak of the material is approximately 105 °C, whereas the onset temperature of thermal degradation determined is approximately 420 °C. Therefore, 130 °C is close to or slightly above the melting range of the crystalline phase but remains substantially below the onset of thermal decomposition.

At 130 °C, part of the crystalline lamellae may melt, and the mobility of molecular-chain segments in the amorphous regions increases. Nevertheless, XLPE is a chemically crosslinked three-dimensional network and does not become a freely flowing melt after the crystalline phase partially melts. The crosslinked network maintains the macroscopic geometry and dimensional stability of the specimens. During the aging experiment, no visible flow, collapse, or loss of specimen shape was observed.

Thus, the specimens at 130 °C are more appropriately described as crosslinked polymer networks containing partially melted crystalline regions rather than as conventional rigid solids or uncrosslinked polymer melts. The selected temperature was used to accelerate thermo-oxidative degradation within a laboratory-feasible period while avoiding thermal decomposition of the polymer matrix.

Nevertheless, temperature acceleration is valid only when the dominant degradation mechanism remains unchanged. The accelerated results are therefore used primarily for comparative assessment among different thermal pre-aging states. Direct conversion into an exact field-service duration requires independently validated activation-energy parameters and field-aging data.

To simulate the service life of submarine cables within a laboratory-feasible timeframe, the experimental duration was scientifically scaled based on chemical kinetics principles. The Arrhenius equation describes the exponential relationship between the chemical reaction rate constant k and absolute temperature T, where the acceleration factor AF can be defined as the ratio of the actual service life to the laboratory accelerated aging life [21]. By solving a system of rate equations at different temperatures, the formula for calculating the acceleration factor was derived as follows:

(1) $$AF=\frac{L_{\mathrm{op}}}{L_{\mathrm{aging}}}=\frac{C/k_{\mathrm{op}}}{C/k_{\mathrm{aging}}}=\frac{A\exp(-E_{a}/k_{B}T_{\mathrm{aging}})}{A\exp(-E_{a}/k_{B}T_{\mathrm{op}})}=\exp\left[\frac{E_{a}}{k_{B}}\left(\frac{1}{T_{\mathrm{op}}}-\frac{1}{T_{\mathrm{aging}}}\right)\right]$$

where *L*_op_ is the actual service life, and *L*_aging_ is the accelerated aging life. *C* is the critical failure performance value; *k*_op_ is the aging rate constant at the actual operating temperature; *k*_aging_ is the aging rate constant at the accelerated aging temperature; *A* is the prefactor; *E*_a_ is the activation energy (the activation energy used in this paper is 120 kJ/mol); *k*_B_ is the Boltzmann constant; *T*_op_ is the actual operating temperature of the cable in service; and *T*_aging_ is the accelerated aging temperature.

The Arrhenius relationship is introduced here only to explain the general principle of temperature acceleration. Because the activation energy and field-aging kinetics of the specific XLPE compound were not independently validated in this study, no exact conversion from laboratory aging time to field-service years was performed.

The specimens were thermally aged for 360, 720, 1080, and 1440 h to establish progressively increasing levels of thermal pre-damage. These durations were selected to generate distinguishable degradation states rather than to represent exact field-service years.

**Figure 2:**
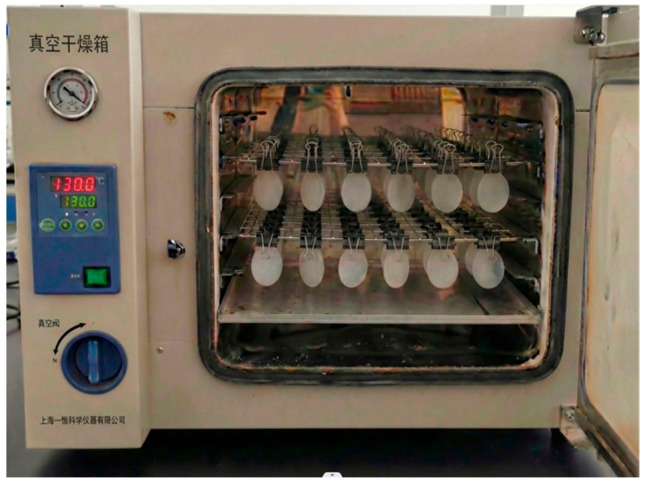
Accelerated thermal aging oven.

The thermal aging test was conducted in a forced-air drying oven. All specimens were suspended vertically on high-temperature-resistant PTFE racks, spaced at least 10 mm apart and kept from contacting the oven walls, as shown in Figure 2. The specimens were retrieved when the preset aging durations of 360 h, 720 h, 1080 h, and 1440 h were reached. The collected specimens were placed in a vacuum desiccator containing color-changing silica gel and stored at room temperature away from light for 24 h to allow for equilibration.

### 2.3. Seawater Erosion Tests on Specimens with Different Degrees of Thermal Aging

Submarine cables face complex corrosion threats during long-term operation in deep-sea environments. Statistical data indicate that mechanical damage caused by human activities accounts for more than 90% of all submarine cable failures, with anchor strikes being the primary threat [22]. When the outer sheath and metal armor of a submarine cable are damaged by anchor strikes, highly saline seawater rapidly penetrates the surface of the XLPE insulation layer. The salinity of seawater along the route of the Third Interconnection Project remains between 30‰ and 34‰ year-round. According to survey data on the carbonate system in the northern shelf region of the South China Sea, among the anions present in seawater, Cl^−^ and SO_4_^2−^ have relatively high concentrations, while HCO_3_^−^ contributes to the carbonate-buffering system. In the present study, these three anions were simultaneously included in the simulated seawater to investigate their combined influence on thermally pre-aged XLPE: the mass concentration of Cl^−^ is approximately 18–19 g/L, that of SO_4_^2−^ is approximately 2.7 g/L, and that of HCO_3_^−^ is approximately 0.14–0.15 g/L [23]. Driven by the electric field and temperature field, these three reactive anions diffuse into the interior of the insulation layer, inducing electrochemical degradation reactions that pose a serious threat to insulation performance.

Because the three anions were not tested separately, the present experimental design does not permit quantitative separation of their individual contributions. Therefore, the results discussed below represent the combined effect of the mixed-anion solution.

The specimens used in this experiment were XLPE materials that had undergone thermal aging treatment. The thermal aging process introduced microstructural changes in the insulating matrix, including reduced crystallinity, increased microporosity, and molecular chain breakage; these structural defects directly affect the subsequent ion penetration pathways and diffusion rates [24]. When seawater corrosion stress was applied to the thermally aged specimens, the sequential evolution of submarine cables—initial thermal damage followed by seawater corrosion—could be realistically replicated, which aligns with actual engineering scenarios. To systematically analyze the time-dependent influence of multi-ion synergistic seawater corrosion on insulation performance, the experiment was designed with four corrosion time points: 168 h, 336 h, 504 h, and 672 h, to capture the dynamic changes in insulation performance at different corrosion stages.

The simulated seawater solution was prepared based on the measured concentrations of the dominant anions in the northern South China Sea. Three active anions—Cl^−^, SO_4_^2−^, and HCO_3_^−^—were contained in the solution with concentrations matching the measured data, thereby providing a controlled mixed-anion environment for evaluating the degradation behavior of thermally pre-aged XLPE under simulated seawater exposure. The specific preparation protocol for the simulated seawater solution was presented in Table 1.

**Figure 3:**
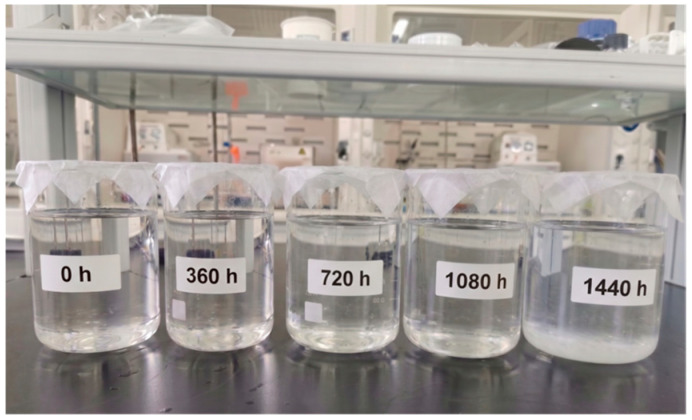
Preparation of the etching solution.

The simulated seawater was prepared by dissolving analytical-grade NaCl, Na_2_SO_4_, and NaHCO_3_ in deionized water at the concentrations listed in Table 1. The solution was prepared volumetrically and thoroughly mixed before use. Five cups of seawater solution are prepared, and XLPE samples with different thermal aging pre-damage time have been distinguished, as shown in Figure 3. 

The seawater corrosion and aging test was conducted in an electric forced-air drying oven. A total of five 1000 mL borosilicate glass beakers were prepared for the experiment. Prior to the start of the experiment, XLPE specimens that had undergone different durations of thermal aging were immersed in the solution-filled beakers to ensure full contact between the specimen surfaces and the solution. To prevent rapid evaporation of moisture at high temperatures, the openings of the beakers were sealed with aluminum foil and high-temperature-resistant plastic wrap, and all beakers were placed on the oven tray.

Thermally pre-aged specimens were fully immersed in 1000 mL of simulated seawater in sealed borosilicate-glass vessels. The solution temperature was maintained at 90 ± 2 °C, as verified by preliminary thermocouple calibration [25].

This seawater corrosion and aging test was conducted at four time points: 168 h, 336 h, 504 h, and 672 h. The maximum duration of 672 h was determined based on statistical data regarding the actual emergency repair cycles for submarine cable projects: according to a statistical report published by the International Council on Large Electric Systems (CIGRE), the average repair time following a submarine cable failure is approximately 60 days, during which the insulation remains continuously exposed to the seawater environment [26]. This experiment was conducted at 90 °C. Compared to the temperature conditions on the deep-sea seabed, both water molecule penetration and ionic chemical corrosion reactions are accelerated at this temperature.

The diffusion behavior of ions in a polymer matrix follows Fick’s second law, which can be expressed in differential form as:

(2) $$\frac{\partial C}{\partial t}=D\frac{\partial^{2}C}{\partial x^{2}}$$

where *C* represents the ion concentration, *t* represents time, *x* represents the distance coordinate along the diffusion direction, and *D* represents the diffusion coefficient.

The diffusion coefficient *D* and temperature *T* satisfy the Arrhenius relationship:

(3) $$D=D_{0}\exp\left(-\frac{E_{d}}{RT}\right)$$

where *D*_0_ is the pre-diffusion factor, *E*_d_ is the diffusion activation energy, *R* is the gas constant, and *T* is the absolute temperature.

Under the experimental condition of 90 °C, the diffusion coefficients of active ions such as Cl^−^ increased significantly.

The maximum exposure duration of 672 h was selected to produce a measurable and progressive corrosion response within a laboratory-feasible period. Because ion diffusion and chemical degradation are temperature dependent, direct time equivalence with field seawater exposure requires independently determined diffusion activation energies and validation under multiple temperatures.

The seawater-exposure temperature of 90 °C was selected as an accelerated condition and does not represent the ambient temperature of deep-sea seawater. It was intended to accelerate water uptake, ion transport, and interfacial degradation in XLPE specimens that had already experienced different levels of thermal pre-damage. This temperature also corresponds to the upper temperature range that may occur in insulation regions close to the conductor during cable operation.

The exposure durations of 168, 336, 504, and 672 h were selected to capture the progressive evolution of dielectric, breakdown, and surface-morphology changes under accelerated conditions. These durations are used as comparative laboratory exposure stages and should not be interpreted as exact equivalents of specific field immersion times.

### 2.4. Statistical Analysis

Broadband dielectric spectroscopy was performed using six independently prepared specimens for each combination of thermal-aging duration and seawater-exposure duration. The dielectric parameters presented in this study correspond to the average values of the repeated measurements. The statistical variation among repeated measurements is illustrated by the error bands shown in the corresponding figures.

AC breakdown tests were conducted using eleven independent specimens under each aging condition. All valid breakdown measurements were included in the two-parameter Weibull analysis to determine the characteristic breakdown field strength and the data dispersion.

SEM characterization was performed on four independently prepared specimens for each selected aging condition. Multiple randomly selected surface regions were examined on each specimen, and only morphological features consistently observed across the independent specimens were regarded as representative.

## 3. Characterization of the Electrical Properties and Microstructure of XLPE Insulation After Stepwise Aging and Corrosion

To systematically evaluate the degradation patterns of the electrical properties of XLPE insulation materials under marine environments and thermal stress, this study established a macro-scale electrical property characterization system based on two dimensions—dielectric response and breakdown failure—and combined it with microscopic morphological analysis to reveal the structural evolution characteristics of the material. Given that polymer materials are highly sensitive to moisture and surface contamination, to minimize the impact of environmental factors on test reproducibility.

Before electrical and SEM characterization, the specimens were briefly rinsed with anhydrous ethanol for 5 s to remove loosely attached salt residues and surface contaminants. The specimens were not immersed in ethanol for an extended period. After rinsing, they were dried at 60 °C for 2 h to reduce the influence of residual free moisture on the electrical measurements. The same pretreatment procedure was applied to all experimental groups.

This procedure was intended to improve the comparability of the electrical measurements by minimizing uncontrolled surface conduction caused by residual seawater salts. However, it may also remove some loosely bound or ethanol-soluble surface products. Therefore, the characterization results reported in this study correspond to the cleaned and dried state of the specimens rather than to the complete surface-deposit state immediately after seawater exposure.

Because the primary objective of this study was to evaluate changes in electrical insulation capability, the characterization framework was centered on dielectric response, breakdown behavior, and the surface microstructural features associated with electrical degradation.

### 3.1. Testing of Relative Permittivity and Tangent of Dielectric Loss Angle

A broadband dielectric spectrometer was used to simultaneously measure the frequency dependence of the dielectric constant and dielectric loss in the range from 0.1 Hz to 1 MHz. For testing, the samples were fabricated into circular discs, and aluminum electrodes were deposited on both the top and bottom surfaces to enhance the adhesion between the metal electrodes and the polymer surface and to reduce measurement drift caused by contact instability. To prevent excessive voltage from causing nonlinear conduction or charge injection effects that could interfere with the small-signal dielectric response, the test voltage was set to 1 V. The frequency range was selected to cover 0.1 Hz to 1 MHz, in order to account for both the contributions of interfacial polarization and conductivity in the low-frequency range and the intrinsic polarization response in the high-frequency range.

The core parameters of broadband dielectric spectrum testing are the relative permittivity ε_r_ and the dielectric loss tangent tan δ. Under parallel-plate electrode conditions, the capacitance and relative permittivity satisfy the following geometric relationship:

(4) $$\varepsilon_{r}=\frac{Cd}{\varepsilon_{0}S}$$

where *C* represents the measured capacitance, *d* represents the sample thickness, *S* represents the effective electrode area, and ε_0_ represents the permittivity of free space. The dielectric loss tangent, tan δ, is either directly provided by the instrument or calculated from the equivalent circuit parameters; it is used to indicate the level of dielectric energy dissipation.

### 3.2. Power–Frequency Breakdown Electric Field Strength Testing and Data Processing

The breakdown field strength is used to characterize the ultimate ability of insulating materials to withstand electric field stress and is an important indicator for evaluating the safety margin of insulation. In this study, power–frequency breakdown tests were conducted using the DDJ-100 kV AC breakdown test system, with the test method following IEC 60243-1:2013 [27]. The test involved a linear increase in 50 Hz AC voltage at a rate of 500 V/s until the specimen broke down, at which point the breakdown voltage was recorded [28]. To suppress air discharge and corona effects, a cylindrical electrode with a diameter of 25 mm was used to contact the specimen, and the breakdown test was conducted in silicone oil. Eleven independent specimens were tested under each aging condition. All valid breakdown measurements were included in the two-parameter Weibull analysis to determine the characteristic breakdown field strength and the statistical dispersion of the data.

The breakdown electric field strength is calculated using the following formula:

(5) $$E_{b}=\frac{U_{b}}{d}$$

where *E*_b_ is the breakdown electric field strength, *U*_b_ is the breakdown voltage, and *d* is the sample thickness.

Since polymer breakdown is a typical statistical failure process, this study uses a two-parameter Weibull distribution to fit the breakdown field strength data [29], whose cumulative distribution function is:

(6) $$F(E)=1-\exp\left[-{\left(\frac{E}{\alpha }\right)}^{\beta }\right]$$

where *α* is the scale parameter, corresponding to the characteristic breakdown electric field strength at a failure probability of 63.2%, and *β* is the shape parameter, reflecting the degree of data dispersion and the consistency of failures. To facilitate parameter regression, it can be linearized as follows:

(7) $$\ln\left[-\ln\left(1-F(E)\right)\right]=\beta \ln(E)-\beta \ln(\alpha )$$

Once *α* and *β* have been obtained through fitting, a unified statistical comparison scale can be established across different aging conditions.

### 3.3. Observation of Microstructural Evolution Using SEM

A SU3500 scanning electron microscope was used to observe and analyze the surface microstructure of XLPE specimens under different aging durations and solution erosion conditions. For SEM observation, the same brief ethanol-rinsing and drying procedure was applied to remove loosely attached salt crystals that could obscure the underlying XLPE surface morphology. Consequently, the SEM images primarily reflect the surface-morphological changes of the polymer matrix after cleaning, rather than the complete distribution of soluble salt deposits and loosely attached corrosion products.

To address the issue of charge accumulation caused by the poor conductivity of XLPE insulation and to improve the clarity and contrast of the secondary electron image, the sample surface was metallized prior to testing. A conductive gold film approximately 10 nm thick was deposited on the sample surface using magnetron sputtering. For SEM observation, the acceleration voltage was set to 15 kV, with a working distance of approximately 10 mm. The magnification was flexibly adjusted according to the scale of microscopic damage features under various aging conditions, with a scanning range covering 500× to 5000×, to balance an overall understanding of the distribution of macroscopic defects with the fine resolution of local damage morphologies such as microcracks and microvoids [30].

A systematic comparison of SEM images of specimens in different states of aging can visually reveal the processes of grain boundary damage, microcrack initiation, and propagation caused by thermal-oxidative aging, as well as microstructural features such as surface corrosion pits and ion channels induced by the penetration of active ions in seawater. This approach effectively links the degradation of macroscopic electrical parameters with the mechanisms of microstructural damage.

### 3.4. Infrared Spectroscopy Characterization Testing

Fourier-transform infrared spectroscopy (FTIR) was employed to characterize the molecular-structure evolution of XLPE and to interpret the degradation of its macroscopic electrical properties from the perspective of chemical-bond changes.

The chemical structures of XLPE specimens subjected to different aging conditions were analyzed using a Nicolet™ iS50 Fourier-transform infrared spectrometer. Before measurement, the specimens were dried in a vacuum oven at 60 °C for 2 h to minimize interference from adsorbed moisture with the spectral baseline and characteristic absorption bands. Spectra were collected in transmission mode over the wavenumber range of 4000–500 cm^−1^ at a spectral resolution of 4 cm^−1^, with 32 scans accumulated for each spectrum to obtain an adequate signal-to-noise ratio. For quantitative peak-area analysis, the transmittance spectra were converted into absorbance spectra, and all spectra were processed using identical baseline-correction and normalization procedures.

During the thermo-oxidative aging of XLPE, methylene groups (–CH_2_–) in the polyethylene backbone may undergo oxidation and chain-scission reactions, resulting in the progressive formation of oxygen-containing polar functional groups. The absorption band centered at approximately 1715 cm^−1^ is mainly assigned to the stretching vibration of carbonyl groups (C=O), whereas the broad band within 3200–3600 cm^−1^ is associated with hydroxyl-group (–OH) stretching vibrations. The absorption bands in the range of 1000–1100 cm^−1^ can be attributed to the stretching vibrations of C–O or C–O–C structures. Variations in these characteristic bands were used to assess the oxidation degree and accumulation of polar functional groups during thermal aging and subsequent seawater exposure.

## 4. Patterns of Performance Degradation Under the Combined Effects of Thermal Aging and Seawater Corrosion

### 4.1. The Evolution of Dielectric Properties Across Different Time Scales

**Figure 4:**
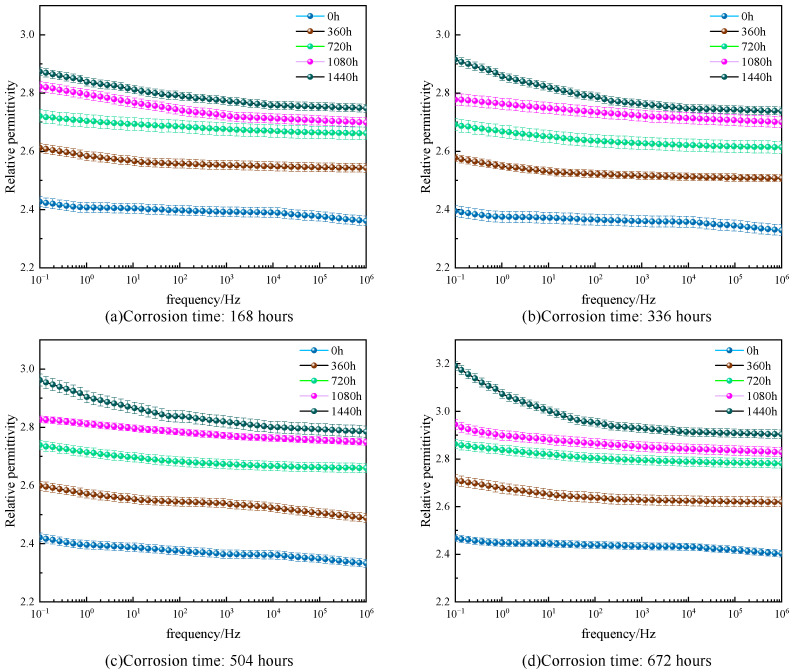
Frequency-dependent relative permittivity of XLPE specimens under different thermal-aging durations and seawater-exposure durations. The solid curves represent the average values obtained from six independent specimens, and the shaded regions indicate the statistical variation of the repeated measurements. (**a**) Corrosion time: 168 h; (**b**) corrosion time: 336 h; (**c**) corrosion time: 504 h; (**d**) corrosion time: 672 h.

**Figure 5:**
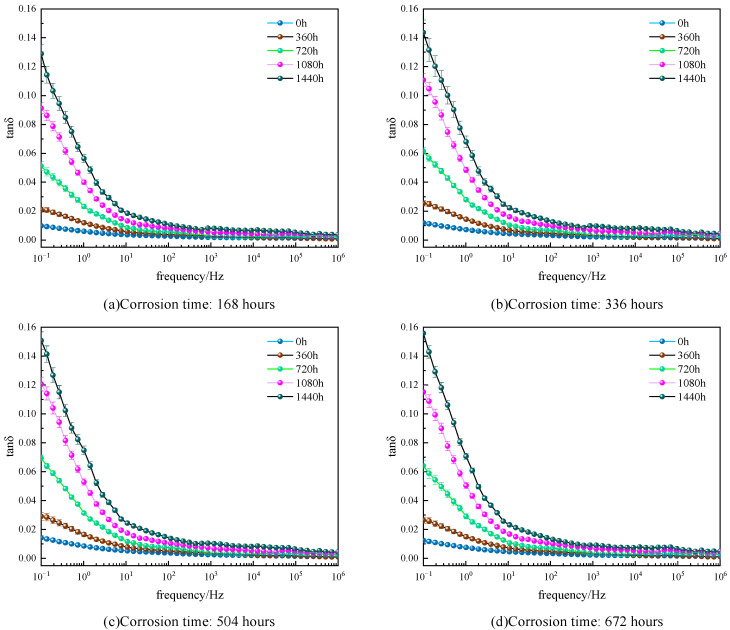
Frequency-dependent dielectric loss tangent of XLPE specimens under different thermal-aging durations and seawater-exposure durations. The solid curves represent the average values obtained from six independent specimens, and the shaded regions indicate the statistical variation of the repeated measurements. (**a**) Corrosion time: 168 h; (**b**) corrosion time: 336 h; (**c**) corrosion time: 504 h; (**d**) corrosion time: 672 h.

For each combination of thermal aging duration and seawater exposure duration, broadband dielectric measurements were performed on six independent specimens. Broadband dielectric measurements were performed on six independent specimens under each aging condition. The dielectric spectra shown in Figure 4 and Figure 5 represent the average values of the repeated measurements, while the shaded error bands illustrate the statistical variation among the six independent specimens. After calculating the average, the dielectric constant and dielectric loss factor for each combination were determined.

Figure 4 and Figure 5 show the frequency-dependent trends of the relative permittivity and dielectric loss factor for various combinations of thermal aging and erosion durations.

Figure 4 shows the variation in the relative permittivity of XLPE insulation specimens over a wide frequency range of 10^−1^ to 10^6^ Hz after undergoing thermal aging for different durations and subsequent seawater exposure for 168 to 672 h. The relative permittivity of all specimens decreases monotonically with increasing frequency and is significantly influenced by both the degree of thermal aging and the duration of seawater exposure, demonstrating that thermal pre-aging increases the susceptibility of XLPE to subsequent seawater-induced dielectric degradation.

The rate of decline in the dielectric constant is significantly higher in the low-frequency range (10^−1^–10^2^ Hz) than in the high-frequency range (10^3^–10^6^ Hz). This is due to differences in the response times of various polarization mechanisms: at low frequencies, both dipole polarization and interfacial polarization can respond fully, whereas at high frequencies, only electronic and atomic polarization are active, causing the dielectric constant to stabilize.

Under the same duration of seawater erosion, the dielectric constant increases monotonically with the duration of thermal aging. Taking the 168 h erosion specimen as an example, the dielectric constant of the 1440 h thermally aged specimen at 1 Hz was 22.3% higher than that of the unaged specimen. The mechanism behind this is that thermal aging generates a large number of polar groups and microscopic defects, thereby enhancing dipole polarization and interfacial polarization effects [31].

At the same degree of thermal aging, the dielectric constant continued to increase with prolonged exposure to seawater, and the higher the degree of thermal aging, the more significant the effect of corrosion. The dielectric constant of the non-aged specimen increased by only 2.9% after 672 h of corrosion, whereas that of the specimen subjected to 1440 h of thermal aging increased by 10.7%. This indicates that the pre-damage caused by thermal aging provides a pathway for the penetration of seawater ions; the accumulation of ions further enhances interfacial polarization [32], creating a positive feedback loop, and provides experimental evidence for subsequent mechanistic analysis.

Figure 5 shows the variation in the dielectric loss tangent (tanδ) of XLPE insulation specimens over a wide frequency range of 10^−1^ to 10^6^ Hz. These specimens were subjected to thermal aging for varying durations to induce pre-damage, followed by seawater exposure for 168 to 672 h. All specimens exhibited the typical characteristic of a high tanδ at low frequencies and a low tanδ at high frequencies. Furthermore, both parameters were significantly influenced by the degree of thermal aging and the duration of seawater exposure, demonstrating a synergistic degradation effect consistent with that observed for the dielectric constant.

tanδ decreases sharply with increasing frequency in the low-frequency range of 10^−1^ to 10^2^ Hz, and tends to level off above 10^3^ Hz. This is because, at low frequencies, dielectric loss is primarily dominated by conductive loss and interfacial polarization loss; at high frequencies, space charge does not have time to migrate, and interfacial polarization cannot be established, leaving only weak dipole relaxation loss. Consequently, tanδ decreases significantly and tends to stabilize.

Under the same seawater exposure time, tanδ increases monotonically with the duration of thermal aging. Taking the 168 h exposure specimen as an example, the tanδ of the 1440 h thermally aged specimen at 0.1 Hz is approximately 0.14, which is more than one order of magnitude higher than that of the non-aged specimen. The mechanism behind this is that the polar groups and microscopic defects generated by thermal aging not only increase the material’s conductive current but also enhance interfacial polarization loss [33].

**Figure 6:**
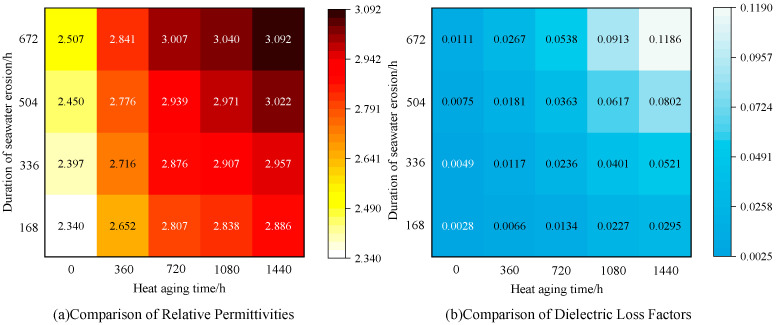
Heat map of power frequency insulation dielectric properties of XLPE specimens under the combined effects of thermal aging and seawater corrosion. (**a**) Comparison of Relative Permittivities; (**b**) comparison of Dielectric Loss Factors.

To more intuitively illustrate the synergistic degradation patterns of the two parameters across the dimensions of thermal aging time and erosion time, Figure 6 presents the distributions of the power frequency dielectric loss factor and relative permittivity under 16 sets of test conditions in the form of a degradation heatmap. The figure shows that both parameters increase monotonically with increasing values in both dimensions, and the color gradient in the regions of high thermal aging and long erosion time is much steeper than that in the upper-left corner. The degradation heatmaps clearly show that dielectric degradation becomes increasingly severe with both thermal aging duration and seawater exposure time. More importantly, the degradation rate increases more rapidly in specimens subjected to severe thermal pre-aging than would be expected from seawater exposure alone, suggesting a strong interaction between the two degradation processes.

Figure 6 shows a heatmap of the dielectric constant and dielectric loss factor of XLPE insulation at a power frequency of 50 Hz as a function of thermal aging and seawater corrosion duration. It provides a visual quantification of the gradient degradation of dielectric properties under the combined effects of these two factors and clearly demonstrates the accelerating effect of pre-damage caused by thermal aging on deterioration due to seawater corrosion.

The dielectric constant heat map shows that the performance degradation exhibits a distinct two-dimensional gradient distribution, increasing monotonically with prolonged thermal aging and seawater exposure. After 672 h of seawater exposure, the dielectric constant of the non-thermally aged specimen was only 2.507, representing a 6.9% increase from the initial value; In contrast, the dielectric constant of the 1440 h thermally aged specimens rose to 3.092 after the same exposure time, representing an increase of 32.1%. The color gradient ranging from light yellow to dark brown indicates that the higher the degree of thermal aging, the more significant the increase in dielectric constant caused by seawater exposure.

The trend in the loss tangent closely follows that of the dielectric constant, but is more sensitive to synergistic effects. For the non-thermally aged specimen, tanδ was only 0.1186 after 672 h of exposure, whereas for the specimen thermally aged for 1440 h, tanδ rose from 0.0295 at 168 h to 0.1186 at 672 h, an increase of 302%. The clear gradient from dark blue to light white clearly indicates that the dielectric loss tangent rises sharply in the regions of high aging degree and long exposure time.

The more pronounced changes in the high-thermal-aging and long-exposure region support a strong dependence of seawater-induced deterioration on thermal pre-damage. This observation is consistent with an interaction between thermally generated defects and subsequent seawater penetration, although the magnitude of a non-additive synergistic contribution cannot be isolated from the present dataset.

### 4.2. Analysis of Thermal Pre-Aging-Dependent Degradation of Breakdown Strength

**Figure 7:**
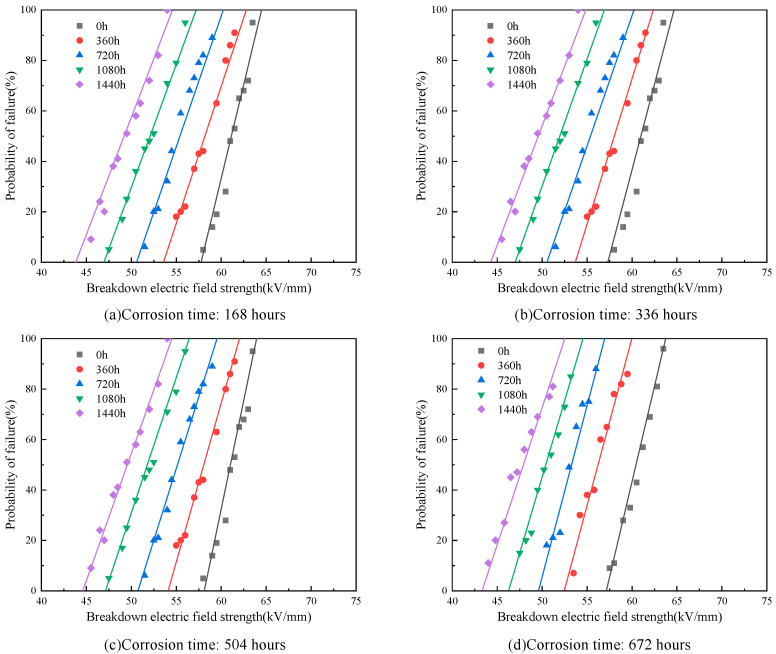
Breakdown field strength distribution of XLPE specimens under the combined effects of thermal aging and seawater corrosion; each Weibull distribution was established from eleven independent breakdown measurements. (**a**) Corrosion time: 168 h; (**b**) corrosion time: 336 h; (**c**) corrosion time: 504 h; (**d**) corrosion time: 672 h.

Eleven specimens were initially tested under each condition. Measurements involving edge flashover, electrode-contact failure, or other predefined non-material breakdown events were classified as invalid and excluded before statistical analysis. Figure 7 shows the probability distribution of AC breakdown field strength failure for four specimens with different thermal aging durations at various erosion times.

The AC breakdown strength under each condition was evaluated using eleven independent specimens. All valid measurements were included in the two-parameter Weibull analysis. The characteristic breakdown strength (α) and the Weibull shape parameter (β) were used to characterize the electrical strength and data dispersion, respectively.

As thermal aging time increases and erosion duration extends, the characteristic breakdown field strength continues to decrease, and the distribution curve shifts overall toward lower field strengths. At an erosion time of 168 h, the characteristic breakdown field strength of each thermal aging group showed a certain degree of reduction compared to the pure thermal aging state, but the decrease was within 10%, indicating that the impact of short-term erosion on breakdown performance is relatively limited, primarily manifested as a slight increase in the density of surface defects. As the erosion duration was extended to 504 h to 672 h, the additional decrease in breakdown field strength increased significantly with the intensification of thermal aging. under the extreme conditions of 1440 h of thermal aging combined with 672 h of erosion, the total reduction in characteristic breakdown field strength compared to the initial value before aging exceeded one-third; compared to specimens with the same degree of thermal aging but subjected to only 168 h of erosion, there was an additional loss of approximately 15%, indicating that the continuous degrading effect of long-term seawater erosion on breakdown performance cannot be ignored.

Figure 7 illustrates the variation in Weibull distribution parameters for the breakdown field strength of XLPE under different combinations of thermal aging and seawater erosion durations. The scale parameter *α* represents the characteristic breakdown field strength corresponding to a 63.2% failure probability, while the shape parameter *β* reflects the dispersion of the breakdown data; a higher *β* value indicates more uniform insulation performance [34].

**Figure 8:**
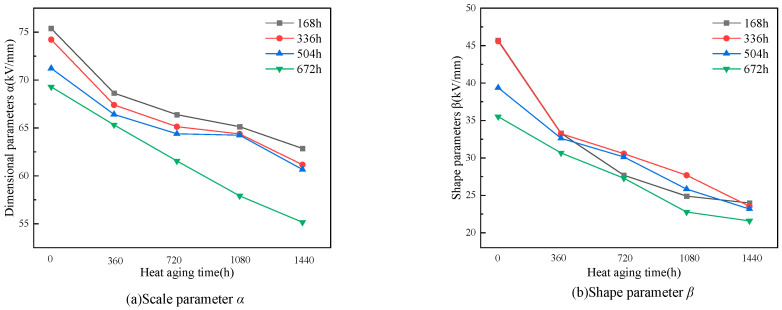
Weibull distribution parameters of the breakdown field strength of XLPE specimens after thermal aging and seawater corrosion. (**a**) Scale parameter α; (**b**) shape parameter β.

Figure 8 shows Weibull distribution parameters of the breakdown field strength of XLPE specimens after thermal aging and seawater corrosion, including scale parameter α and shape parameter β. Overall, both *α* and *β* decrease monotonically with increasing thermal aging time. Furthermore, for the same degree of thermal aging, the longer the seawater exposure time, the more significant the decrease in these parameters. After 672 h of seawater exposure, the *α* value of the non-thermally aged specimen decreased from 75.37 kV/mm to 69.28 kV/mm, a decrease of 8.08%; In contrast, after the same exposure time, the *α* value of the 1440 h thermally aged specimens decreased from 62.84 kV/mm to 55.14 kV/mm, representing a decline of 12.25%, indicating that the pre-damage caused by thermal aging significantly accelerated the degradation of breakdown strength.

The simultaneous decrease in *β* values indicates that the number of internal defects in XLPE increases and becomes unevenly distributed during the aging process, leading to greater variability in insulation performance. Microscopic cracks and polar groups generated by thermal aging provide pathways for the penetration of seawater ions. These ions accumulate at the defects, creating localized high electric fields that further induce the growth of treeing, ultimately contributing to the concurrent deterioration of breakdown strength and data uniformity under the combined aging condition.

### 4.3. The Relationship Between Microstructural Changes and Macroscopic Electrical Properties

To elucidate the damage mechanisms caused by the synergistic effects of thermal aging and seawater corrosion on XLPE insulation specimens at the microscopic level, and to further explain the material–structural origins of the deterioration in macroscopic electrical parameters, this section presents scanning electron microscopy (SEM) analyses of XLPE specimens that underwent a 1440 h thermal aging process at various stages, followed by exposure to seawater for varying durations. The results provide microscopic evidence for the aforementioned patterns of electrical performance degradation based on surface morphology and damage.

SEM observations were performed on four independently prepared specimens for each selected aging condition. Multiple randomly selected surface regions were examined on each specimen, and only morphological features consistently observed across the independent specimens were considered representative.

**Figure 9:**
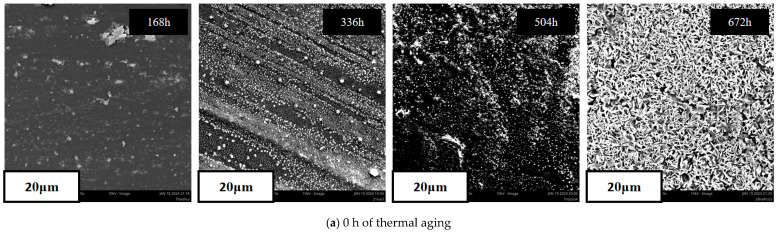

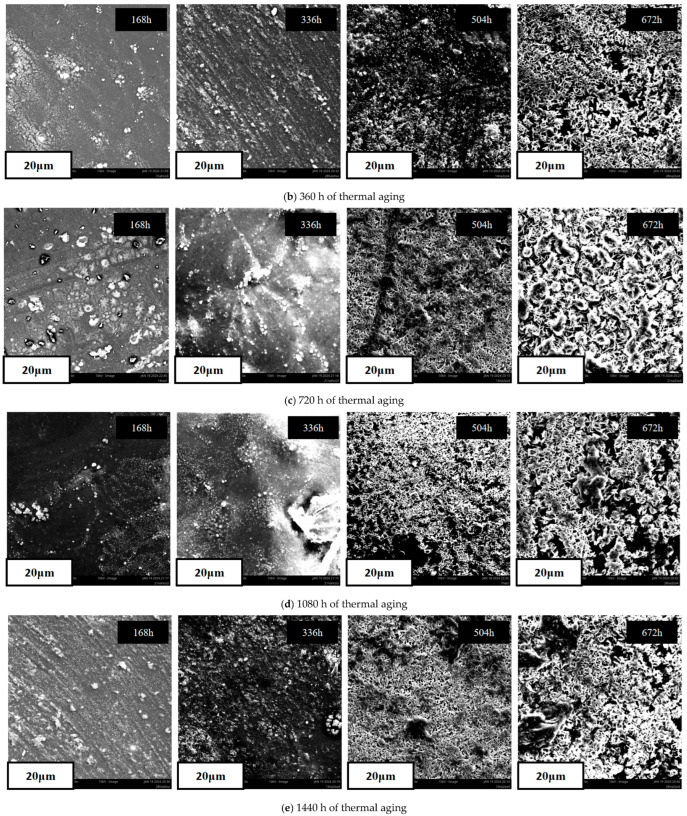
Changes in the surface morphology of XLPE specimens after different durations of thermal aging and seawater corrosion, representative SEM images selected from four independently prepared specimens: (**a**) 0 h of thermal aging; (**b**) 360 h of thermal aging; (**c**) 720 h of thermal aging; (**d**) 1080 h of thermal aging; (**e**) 1440 h of thermal aging.

Figure 9 shows the evolution of the surface microstructure of XLPE specimens after exposure to simulated seawater for 168 h, 336 h, 504 h, and 672 h, following treatment with different thermal aging durations. Figure 9 shows the evolution of the surface morphology of XLPE specimens after different thermal aging and exposure durations.

Figure 9 shows the evolution of the surface microstructure of XLPE specimens with varying degrees of thermal aging-induced pre-damage after 168–672 h of seawater exposure, providing a clear illustration of the microscopic processes involved in the synergistic interaction between thermal aging and seawater erosion.

The surface of the unaged sample was initially dense and smooth; after 168 h of exposure, only a few tiny particles appeared, and a uniform corrosion layer did not form until 672 h, indicating that pure XLPE exhibits good resistance to seawater corrosion. As the heat aging duration increased, surface defects on the specimens gradually increased. Specimens heat-aged for 360 h exhibited noticeable corrosion spots after 168 h of erosion, while specimens heat-aged for 720 h or more showed cracks and pinholes on their surfaces after 336 h of erosion.

After 672 h of corrosion, the surface of the 1440 h thermal aging specimen exhibited severe honeycomb-like corrosion structures, and the material matrix had been extensively damaged. This morphological evolution closely aligns with the degradation patterns of dielectric properties and breakdown strength. Defects generated by thermal aging, along with polar groups, provide pathways for the penetration of seawater ions. Ions accumulate at the interface, enhancing interfacial polarization, which leads to an increase in the dielectric constant and dielectric loss; simultaneously, localized high electric fields form at the defects, inducing the growth of tree-like structures, ultimately causing a simultaneous decline in breakdown strength and insulation uniformity, and these observations are consistent with the interpretation that thermal-aging-induced surface defects increase the susceptibility of XLPE to subsequent seawater-related deterioration. Nevertheless, the SEM observations provide morphological support for this interaction rather than quantitative proof of a non-additive synergistic effect.

### 4.4. FTIR Analysis of Molecular-Structure Evolution

To elucidate the seawater-induced damage mechanism of XLPE insulation at the molecular level and to clarify the molecular–structural basis for the deterioration of macroscopic electrical properties, FTIR analysis was performed on XLPE specimens thermally aged for 1440 h and subsequently exposed to simulated seawater for 0, 168, 336, 504, and 672 h. The spectroscopic results were used to provide chemical-structure evidence complementary to the dielectric, breakdown, and surface-morphology observations discussed above.

**Figure 10:**
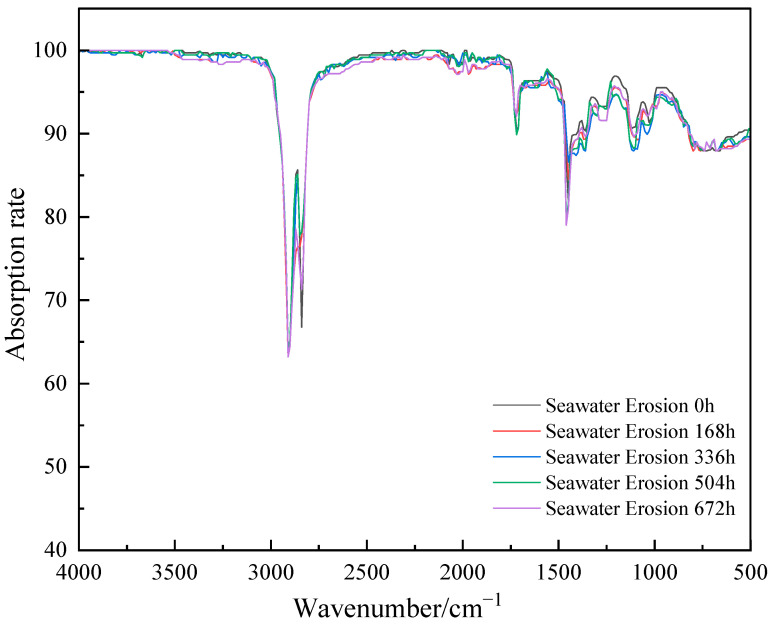
Comparison of FTIR spectra of unaged XLPE specimens and 1440 h thermally aged XLPE specimens under different seawater erosion durations.

To focus on the most representative severe pre-damage condition, specimens subjected to 1440 h of thermal aging were selected for detailed characterization. Figure 10 compares the FTIR spectra of the unaged specimen with those of the thermally pre-aged specimens after different seawater-exposure durations. All specimens exhibited the characteristic absorption bands of the polyethylene backbone at approximately 2910, 2850, 1460, and 720 cm^−1^, which are assigned to the asymmetric stretching, symmetric stretching, scissoring, and rocking vibrations of methylene groups (–CH_2_–), respectively. Variations in these methylene-related bands provide information on changes in the polyethylene backbone during sequential aging.

Because Figure 10 is presented in transmittance, a lower transmittance at an absorption band corresponds to stronger infrared absorption. For quantitative comparison, the transmittance spectra were converted into absorbance spectra and processed using identical baseline-correction, normalization, and peak-integration procedures. The normalized methylene-related band indices showed an overall decrease with increasing seawater-exposure duration, and the lowest values were observed for the specimen exposed for 672 h. This evolution is consistent with the progressive disruption of methylene-containing chain segments and the accumulation of molecular-chain damage during prolonged seawater exposure.

In the carbonyl region of 1700–1750 cm^−1^, a carbonyl-related absorption band was detected near 1715 cm^−1^ in the sequentially aged specimens. Because all seawater-exposed specimens had undergone the same 1440 h thermal pre-aging treatment, the absence of a pronounced further increase in this band from 168 to 672 h suggests that seawater exposure caused only limited additional accumulation of carbonyl-containing oxidation products under the present experimental conditions. This result indicates that the chemical changes occurring during the seawater-exposure stage cannot be explained solely by continued carbonyl-forming oxidation. Nevertheless, FTIR alone does not identify the complete reaction pathway or exclude the formation of other oxygen-containing species.

Within the range of 3200–3600 cm^−1^, the broad hydroxyl-related absorption band became progressively more pronounced with increasing seawater-exposure duration. This change indicates an increase in hydroxyl-containing species and/or strongly bound water and is consistent with increased polarity, hydrophilicity, and hydrogen-bonding interactions in the aged XLPE matrix. These changes may enhance moisture affinity and interfacial polarization, thereby contributing to the observed increase in relative permittivity and dielectric loss.

### 4.5. Limitation Analysis of the Obtained Experimental Results

The present accelerated experiments reproduce the sequence of thermal pre-aging followed by seawater ingress at the material level, but they do not fully reproduce the service environment of a complete submarine cable. In actual cables, XLPE insulation is subjected to radial temperature gradients, electric fields, hydrostatic pressure, mechanical constraint, limited oxygen transport, and interactions with semiconductive screens and metallic layers. These factors may alter oxidation kinetics, water uptake, ion migration, and defect development.

Therefore, the present results should be interpreted primarily as comparative evidence of how prior thermal aging modifies the subsequent seawater sensitivity of XLPE. The results establish degradation trends and mechanistic relationships under controlled accelerated conditions, but direct extrapolation to field-service time or full-cable remaining life requires multi-temperature validation, full-scale cable tests, and long-term service data.

## 5. Study on the Microstructural Mechanisms of Synergistic Aging Due to Thermal Aging and Seawater Corrosion

The experimental results indicate that prior thermal aging increases the susceptibility of XLPE to subsequent seawater-induced degradation. The following discussion presents possible material-level explanations consistent with the observed electrical and microstructural evolution. To elucidate the underlying mechanisms of this synergistic degradation process, this section will conduct an in-depth analysis at the microscopic level, focusing on molecular chain scission, the formation of polar groups, and the diffusion of ions.

### 5.1. Microstructural Mechanisms of Thermal Pre-Aging-Dependent Seawater-Induced Degradation

The thermal aging of XLPE is essentially a thermal oxidation-induced free-radical chain reaction [35], which primarily consists of four stages: initiation, growth, branching, and termination. This process is accompanied by molecular chain breakage and the restructuring of cross-linked networks, ultimately resulting in the formation of a large number of polar groups and microscopic defects.

Under the influence of heat, the C–H bonds—which have the weakest bond energy in the XLPE molecular chain—undergo homolytic cleavage, releasing hydrogen atoms and forming alkyl radicals. This is the initial step in the thermal oxidation chain reaction, providing the initial active sites for the subsequent oxidation process.

(8) $$-{\left({\mathrm{CH}}_{2}-{\mathrm{CH}}_{2}\right)}_{n}\overset{Hot}{\to }-{\left({\mathrm{CH}}_{2}-\dot{C}H\right)}^{-}+H\cdot$$

Highly reactive alkyl radicals can rapidly undergo addition reactions with oxygen in the system, forming peroxy radicals. These products are the core active species in the chain transfer stage and serve as the key intermediate radicals that drive the thermal oxidation reaction forward.

(9) $$-{\left({\mathrm{CH}}_{2}-\dot{C}H\right)}^{-}+O_{2}\to -{\left({\mathrm{CH}}_{2}-\mathrm{CH}(\mathrm{OO}\cdot )\right)}^{-}$$

Peroxy radicals abstract hydrogen atoms from adjacent XLPE molecular chains, forming stable hydroperoxides (ROOH) while simultaneously generating new alkyl radicals. This process drives the propagation of the chain reaction and constitutes the core reaction pathway of the chain growth stage. Hydroperoxides have poor thermal stability; under sustained heat, the O-O bonds within their molecules undergo homolytic cleavage, decomposing into alkoxy radicals and hydroxyl radicals. These two types of radicals can trigger further chain reactions, significantly accelerating the thermal aging process of materials.

(10) $$-{\left({\mathrm{CH}}_{2}-\mathrm{CH}(\mathrm{OO}\cdot )\right)}^{-}+-{\left({\mathrm{CH}}_{2}-{\mathrm{CH}}_{2}\right)}^{-}\to -{\left({\mathrm{CH}}_{2}-\mathrm{CH}(\mathrm{OOH})\right)}^{-}+-{\left({\mathrm{CH}}_{2}-\dot{C}H\right)}^{-}$$

(11) $$-{\left({\mathrm{CH}}_{2}-\mathrm{CH}(\mathrm{OOH})\right)}^{-}\overset{Hot}{\to }-{\left({\mathrm{CH}}_{2}-\mathrm{CH}(O\cdot )\right)}^{-}+\cdot \mathrm{OH}$$

Alkoxy radicals readily undergo β-scission reactions, which directly cause the breaking of chemical bonds in the XLPE molecular backbone, resulting in a decrease in molecular weight. At the same time, this process generates polar functional groups such as carbonyl groups (C=O), which are major contributors to the deterioration of the material’s dielectric and mechanical properties.

(12) $$-({\mathrm{CH}}_{2}-{\mathrm{CH}}_{2}-\mathrm{CH}(O\cdot ))-{\mathrm{CH}}_{2}^{-}\to -({\mathrm{CH}}_{2}-\mathrm{CHO})+\cdot {\mathrm{CH}}_{2}-{\mathrm{CH}}_{2}^{-}$$

Highly reactive hydroxyl radicals can abstract hydrogen atoms from XLPE molecular chains, reacting to form water molecules and new alkyl radicals. This process not only sustains the chain-level oxidation reaction but also causes the generated water to further degrade the material’s insulating properties. Subsequently, when two alkyl radicals encounter each other, a coupling termination reaction occurs, causing them to combine and form new C-C cross-links. This reaction restructures the cross-linked network of XLPE and alters the material’s crystallinity, representing a key pathway in the structural evolution during the aging process.

(13) $$\cdot \mathrm{OH}+-{\left({\mathrm{CH}}_{2}-{\mathrm{CH}}_{2}\right)}^{-}\to H_{2}O+-{\left({\mathrm{CH}}_{2}-\dot{C}H\right)}^{-}$$

(14) $$2\cdot {\mathrm{CH}}_{2}{{\text{-}\mathrm{CH}}_{2}}^{-}\to -{\left({\mathrm{CH}}_{2}{\text{-}\mathrm{CH}}_{2}{\text{-}\mathrm{CH}}_{2}{\text{-}\mathrm{CH}}_{2}\right)}^{-}$$

when two peroxy radicals come into contact, they undergo a dismutation or coupling reaction, producing stable, inert products and releasing oxygen. This process consumes the active free radicals in the system and ultimately terminates the chain oxidation reaction, making it the primary termination mechanism in thermal aging.

(15) $$2\mathrm{ROO}\cdot \to \mathrm{ROOR}+O_{2}$$

These reactions ultimately lead to chain breakage, resulting in a decrease in molecular weight and a reduction in crosslinking density. They also generate a large number of polar groups, significantly increasing the dielectric constant and dielectric loss. The volatilization of small-molecule byproducts creates microporosity and cracks, which serve as pathways for the penetration of seawater ions and simultaneously lower the breakdown voltage.

### 5.2. Mechanisms of the Synergistic Effects of Thermal Aging and Seawater Corrosion

The corrosive effects of the three major anions in seawater (Cl^−^, SO_4_^2−^, and HCO_3_^−^) on XLPE occur primarily through three mechanisms: ionic permeation, catalytic oxidation, and chemical degradation [36], all of which exhibit strong synergistic effects with the polar groups and microscopic defects generated by thermal aging.

Cl^−^ was the most concentrated anion in the simulated seawater used in this study and may therefore make an important contribution to ion transport and charge accumulation in XLPE. Thermally generated microvoids and cracks may facilitate its penetration into the polymer matrix. However, because Cl^−^, SO_4_^2−^, and HCO_3_^−^ were present simultaneously and were not evaluated using separate control solutions, the specific contribution of Cl^−^ cannot be isolated from the present results. Cl^−^ rapidly penetrates into the interior of XLPE through microcracks and free voids generated by thermal aging, and migrates directionally under the influence of an electric field, resulting in ionic conductivity:

(16) $$\mathrm{NaCl}\to {\mathrm{Na}}^{+}+{\mathrm{Cl}}^{-}$$

Cl^−^ can undergo a nucleophilic substitution reaction with hydroperoxides generated by thermal aging, producing highly reactive radicals that significantly accelerate the rate of oxidation:

(17) $$\mathrm{ROOH}+{\mathrm{Cl}}^{-}\to \mathrm{RO}\cdot +{\mathrm{OH}}^{-}+\mathrm{Cl}\cdot$$

(18) $$\mathrm{Cl}\cdot +-{\left({\mathrm{CH}}_{2}-{\mathrm{CH}}_{2}\right)}^{-}\to \mathrm{HCl}+-{\left({\mathrm{CH}}_{2}-\dot{C}H\right)}^{-}$$

The generated HCl further lowers the local pH of the material, accelerating molecular chain breakage under acidic conditions; at the same time, HCl itself acts as a proton donor in oxidation reactions.

SO_4_^2−^ has a larger ionic radius and exhibits weaker direct penetration capabilities than Cl^−^, but it primarily generates highly reducing substances through electrochemical reduction, thereby damaging the molecular structure of XLPE. Under the influence of a high electric field, SO_4_^2−^ can undergo a reduction reaction with the cathode to form sulfate radical anions:

(19) $${{\mathrm{SO}}_{4}}^{2-}+e^{-}\to {{\mathrm{SO}}_{4}}^{-}\cdot$$

The highly reactive free radicals generated can abstract hydrogen atoms from the XLPE molecular chains, triggering new oxidative chain reactions.

Through hydrolysis, HCO_3_^−^ creates a slightly alkaline local environment, causing the hydrolysis of ester bonds in XLPE; at the same time, its buffering effect helps sustain the ongoing oxidative reaction.

(20) $${\mathrm{HCO}}_{3}^{-}+H_{2}O⇌H_{2}{\mathrm{CO}}_{3}+{\mathrm{OH}}^{-}$$

OH^−^ attacks the ester bonds formed as byproducts during the XLPE cross-linking process, as well as the ester groups generated by thermal oxidation, causing an alkaline hydrolysis reaction that leads to chain breakage:

(21) $${R\text{-}\mathrm{COO}\text{-}R}^{\prime }+{\mathrm{OH}}^{-}\to {R\text{-}\mathrm{COO}}^{-}+R^{\prime }\text{-}\mathrm{OH}$$

The buffer system formed by HCO_3_^−^ and CO_3_^2−^ neutralizes the acidic byproducts of the oxidation reaction, preventing the local pH from dropping too low and inhibiting the reaction rate, thereby ensuring that thermal oxidative degradation proceeds continuously and stably.

In summary, the synergistic aging of XLPE caused by thermal aging and seawater corrosion is essentially a positive feedback loop involving thermal-oxidative pre-damage and accelerated ion penetration. Thermal aging first triggers molecular chain breakage, generating polar groups and microscopic defects that serve as penetration pathways for seawater ions; incoming anions such as Cl^−^ catalyze the decomposition of hydroperoxides, accelerating free radical chain reactions and further damaging the molecular structure. This cycle continuously exacerbates the evolution of microscopic defects, ultimately leading to the deterioration of dielectric properties and a decrease in breakdown strength.

## 6. Conclusions

This study investigated the degradation behavior of XLPE submarine cable insulation subjected to sequential thermal pre-aging and simulated seawater exposure. By combining broadband dielectric spectroscopy, AC breakdown testing with Weibull analysis, SEM observation, and FTIR characterization, the influence of prior thermal-aging damage on subsequent seawater-induced deterioration was systematically evaluated. The main conclusions are summarized as follows:

1. The electrical degradation behavior of XLPE insulation during seawater exposure was strongly dependent on the prior thermal-aging state. With increasing thermal pre-aging duration and seawater exposure time, the relative permittivity and dielectric loss tangent continuously increased, whereas the characteristic breakdown field strength and Weibull shape parameter decreased. Under the most severe condition of 1440 h thermal pre-aging followed by 672 h seawater exposure, the power–frequency relative permittivity increased by 32.1%, while the characteristic breakdown field strength decreased by more than one-third compared with the initial state. These results demonstrate that thermal pre-aging significantly increases the sensitivity of XLPE insulation to subsequent seawater-induced electrical deterioration.
2. The deterioration of electrical properties was closely associated with molecular and microstructural evolution of the XLPE matrix. Thermal aging promoted molecular-chain oxidation, formation of polar functional groups, and generation of microvoids and cracks, which provided preferential pathways for subsequent water and ion penetration. During seawater exposure, these pre-existing defects enhanced interfacial polarization, charge accumulation, and defect-assisted electrical degradation. SEM observations revealed progressive development of pores, cracks, corrosion pits, and honeycomb-like surface structures, while FTIR analysis confirmed the evolution of molecular-chain damage and hydroxyl-related species during sequential aging.
3. The present study provides material-level experimental evidence demonstrating that the degradation response of XLPE insulation under seawater exposure is governed by its previous thermal-aging history. The results highlight the importance of considering accumulated thermal damage in the condition assessment of submarine cable insulation. However, the current accelerated laboratory tests do not establish a direct lifetime prediction model, and the individual contributions of different seawater ions cannot be quantitatively separated. Future studies should incorporate ion-specific exposure experiments, multi-temperature aging tests, mechanical-property evaluation, extruded cable specimens, and full-scale validation to further improve the reliability of degradation assessment models.

Several limitations should be noted. The current dataset does not provide mathematical proof of a non-additive synergistic effect because complete matched thermal-only, seawater-only, and combined-aging control groups were not included. The compression-molded specimens do not fully reproduce the extrusion-induced morphology, crystallinity distribution, residual stress, and multilayer structure of industrial submarine cables. In addition, seawater exposure at 90 °C was used as an accelerated laboratory condition and does not directly represent the ambient deep-sea environment. Therefore, the present findings should be interpreted as comparative material-level degradation evidence rather than an exact representation of field-service behavior. This study also does not establish a direct remaining-service-life prediction model because no failure threshold or long-term degradation law was developed, and mechanical properties were not evaluated. Future work should include ion-specific controls, multi-temperature exposure tests, physicochemical and mechanical characterization, extruded cable specimens, and full-scale cable validation.

## Figures and Tables

**Table 1:** Protocol for Preparing Simulated Seawater Solutions.

Solution Name	Chemical Reagents	Dosage (mg/L)	Target Anion	Anion Concentration (g/L)
Seawater solution	NaCl	3.048 × 10^4^	Cl^−^	18.50
	Na_2_SO_4_	3.92 × 10^3^	SO_4_^2−^	2.65
	NaHCO_3_	2.0 × 10^2^	HCO_3_^−^	0.145

## Data Availability

Due to the fact that the data are related to ongoing follow-up studies, the data from this study will not be made publicly available at this time. If other researchers have a legitimate need for the data, please contact the corresponding author to obtain them.
